# Knowledge and attitudes towards performing resuscitation among seniors - a population-based study

**DOI:** 10.1186/s13690-024-01301-9

**Published:** 2024-05-08

**Authors:** Tomasz Kłosiewicz, Sandra Śmigasiewicz, Hanna Cholerzyńska, Wiktoria Zasada, Adam Czabański, Mateusz Puślecki

**Affiliations:** 1 Department of Medical Rescue, Faculty of Health Sciences, Poznan University of Medical Sciences, 7 Rokietnicka Street, Poznań, 60-608 Poland; 2 Faculty of Health Sciences, Poznan University of Medical Sciences, 7 Rokietnicka Street, Poznań, 60- 608 Poland; 3 Faculty of Administration and National Security, The Jacob of Paradies University, 52 Fryderyka Chopina Street, Gorzów Wielkopolski, 66-400 Poland

**Keywords:** Cardiopulmonary resuscitation, Older adults, Bystander, CPR knowledge

## Abstract

**Background:**

Cardiac arrest constitutes a critical medical emergency necessitating swift intervention to reinstate normal heart rhythm and prevent harm to vital organs. The pivotal role of bystander cardiopulmonary resuscitation (CPR) in influencing survival rates is well recognized. With older adults being the most common group to witness such events, it’s curcial to understand their attitudes and knowledge about performing CPR. Additionally, understanding if health status has an influence can help in tailoring education for specific seniors needs.

**Methods:**

A cross-sectional survey was sent to University of the Third Age (UTA) students. The survey comprised sections focusing on demographic data, CPR knowledge, automated external defibrillator (AED) knowledge, first aid training, and readiness to perform CPR and use AEDs. Participants’ health conditions were also assessed through multiple-choice options.

**Results:**

We received 456 responses. Significant awareness of emergency numbers and cardiac arrest recognition was revealed. However, knowledge gaps persisted, particularly in compression rates. Most participants comprehended AED usage, yet training primarily relied on theoretical approaches. Health conditions notably affected CPR readiness, with associations between specific chronic diseases and willingness to perform CPR.

**Conclusions:**

Addressing knowledge gaps and tailoring education for elderly needs are crucial for improving survival rates. Future research should explore barriers to bystander CPR during out-of-hospital cardiac arrests to further enhance survival prospects.

**Supplementary Information:**

The online version contains supplementary material available at 10.1186/s13690-024-01301-9.

**Table:** 

Text box 1. Contributions to the literature
- There is a limited amount of literature on senior’s knowledge and attitude toward CPR despite elderly being the most common age group to witness cardiac arrest.
- The presence of chronic conditions was related to a lower willingness to perform CPR in polish seniors.
- More than half of our study group received first aid training over 10 years ago or had never received it, which underlines the urgent need for educational programs targeting seniors.

## Background

Cardiac arrest represents a life-threatening medical emergency necessitating immediate intervention to reinstate the heart’s rhythm and prevent irreversible harm to vital organs. The efficacy of resuscitation endeavors substantially hinges upon the swift commencement of bystander cardiopulmonary resuscitation (CPR) prior to the arrival of professional medical aid. Frequently, the willingness and capacity of individuals to engage in CPR assume a pivotal role in determining the survival prospects of victims [1, 2]. Out-of-hospital cardiac arrests (OHCA) predominantly occur in domestic settings, where elderly bystanders are often the first ones to initiate CPR. Notably, the prevalence of bystander CPR and subsequent survival rates following OHCA tend to be diminished in private residences relative to public venues [3].

Previous research has demonstrated substantial variability in the general population’s knowledge of CPR and their willingness to administer it across diverse countries and age groups. As indicated by the EuReCa TWO Study, bystanders initiated CPR in 58% of cases, with this percentage ranging from 13 to 82% [4]. Additionally, in other nations, elderly laypersons exhibited inferior skills and limited knowledge relative to their younger counterparts [5, 6]. This disparity underscores existing gaps in knowledge and misconceptions pertaining to resuscitation, which may impede swift and efficacious responses during cardiac emergencies. Nonetheless, scant information is available regarding the specific attitudes and beliefs held by Polish seniors concerning resuscitation.

As the population ages, older adults frequently confront a multitude of health challenges that can potentially impede their capacity to respond adeptly during a cardiac emergency [7]. Age-related health conditions, including chronic diseases, mobility constraints, cognitive impairments, and frailty, are common factors that may hinder the prompt initiation of CPR [8]. These health circumstances can influence seniors’ perceptions of their own physical capabilities and self-efficacy in executing life-saving maneuvers, consequently impacting their willingness to intervene during emergency situations [9]. Therefore, health status may play a crucial role in determining preparedness to perform CPR, making it essential to examine the impact of health conditions on seniors’ readiness to respond in critical situations.

CPR training programs typically prioritize the general population, often disregarding the distinct requirements and apprehensions of seniors [10]. By identifying knowledge gaps and misconceptions among the elderly concerning CPR, tailored educational initiatives can be formulated. The dissemination of accurate CPR information, coupled with the rectification of misconceptions, has the potential to empower seniors to actively engage in resuscitation efforts, thereby potentially augmenting the prospects of victim survival. Consequently, comprehending the knowledge and attitudes of the elderly towards resuscitation stands as a pivotal necessity to enhance survival rates and to design targeted educational interventions aimed at bolstering bystander CPR rates.

Our objective was not only to collect information on seniors’ readiness to perform CPR but also to understand how this readiness potentially interacts with comorbid healthcare conditions and individual characteristics of the participants. By examining these factors, we aimed to gain a comprehensive understanding of the challenges and potential factors that influence resuscitation readiness within the elderly population.

## Materials and methods

### Study design and data collection methods

The research utilized a cross-sectional survey study design to investigate knowledge and preparedness for CPR and automated external defibrillator (AED) use among students of Universities of the Third Age (UTA). The survey instrument was developed by the authors and consisted of 21 questions covering various aspects related to CPR, AED, first aid training, and reasons for unwillingness to participate. Additionally, respondents were asked about any chronic conditions they suffered from, with multiple-choice options provided.

The survey comprised five sections, with each section containing questions pertaining to specific themes: (1) basic epidemiological data, (2) CPR knowledge, (3) knowledge about AED, (4) first aid training, and (5) preparedness for CPR and AED use, along with reasons for unwillingness to participate. A separate question allowed respondents to indicate any chronic conditions they had, with multiple-choice options provided, such as heart failure, anemia, urinary incontinence, rheumatoid arthritis, diabetes mellitus, depression, atherosclerosis, osteoporosis, and vertigo.

The questionnaire was pretested twice with 10 participants in the first round and 15 participants in the second round, all from the same demographic group as the target population - students of Universities of the Third Age. Minor adjustments were made based on their feedback to improve the questionnaire’s clarity and relevance.

The survey can be found in Supplementary Material.

### Sample characteristics and survey administration

The study population comprised students from Polish UTA. Convenience sampling was used to include participants who voluntarily took part in the survey. The sample size was determined based on the total number of eligible students. According to the Polish Statistical Office there were 113,200 students at UTA. Assuming a confidence level of 95%, the sample size was calculated at 383 respondents. The questionnaire was created using Google Forms and distributed to the participants through an online survey link. The participants were given access to the survey link during two weeks span.

### Ethical considerations

The survey was waived approval from the Institutional Review Board and followed the principles of Good Clinical Practice. Participants provided informed consent before participating. Survey responses were kept anonymous, and no personally identifiable information was collected. Data were stored securely with restricted access to authorized researchers only, ensuring confidentiality and preventing unauthorized access.

### Statistical analysis

The data were processed using Microsoft Excel (Microsoft Corporation, Redmond, Washington, USA) Statistica v12 was used for statistical analysis (Tibco Software Inc., Palo Alto, California, USA). First, the quantitative variables were checked for normality with the use of the Shapiro-Wilk W test. As they did not satisfy normal distribution criteria, they were expressed as median [interquartile range (IQR)]. The categorical variables were expressed as absolute values (n) and absolute frequencies (%). The chi-square test was utilized to evaluate significant differences among the analyzed qualitative variables. A Likert scale was used to describe the questionnaire answers concerning subjective evaluation of health condition (1 – very poor health condition, 5 – very good healt condition). A nonparametric Kruskal‒Wallis test was conducted to investigate differences between more than two groups. The results were considered statistically significant at *p* < 0.05.

## Results

### Study group

Four hundred and fifty-six individuals took part in our study. A majority of the study group consisted of women (87.71%, *n* = 400). Median age was 68 [66–70] years old. The youngest participant was 65 and the oldest 89 years old. Almost all respondents had post-secondary education - university or high school (49.12% *n* = 224 and 42.98%, respectively, *n* = 196). The distribution of respondent’s place of residence was similar. Inhabitants of rural areas constituted the smallest group (14.06%, *n* = 60), while citizens of large cities constituted the largest group (26.31%, *n* = 120). The vast majority of respondents were in the 65–70 age range. This group represented 76.31% (*n* = 348) of the study group. The detailed distribution of epidemiological characteristics was presented in Table 1. The details concerning health condition were summarized in Table 2.

**Table 1:** The detailed characteristics of the study group

Variable		*n*	%
*Marital status*			
	divorced	60	13.15
	married	272	59.64
	widowed	100	21.92
	single	24	5.26
*Level of Education*			
	tertiary	224	49.12
	secondary	196	42.98
	primary	20	4.38
	basic vocational	16	3.51
*Side of living*			
	countryside	64	14.03
	city < 20,000 inhab.	108	23.68
	city 20,001-100,000 inhab.	92	20.17
	city 100,001-500,000 inhab.	72	15.79
	city > 500,000 inhab.	120	26.31

**Table 2:** Subjective assessment of health status and chronic diseases among study participants

Health condition (Likert scale)		*n*	%
	1 – very poor health condition	0	0.00
	2	28	6.14
	3	152	33.33
	4	216	47.36
	5– very good health condition	60	13.15
*Chronic diseases*			
	heart failure	64	14.03
	anemia	32	7.01
	urinary incontinence	84	18.42
	rheumatoid arthritis	56	12.28
	diabetes mellitus	76	16.67
	depression	44	9.65
	atherosclerosis	64	14.03
	osteoporosis	36	7.89
	vertigo	112	24.56

### Descriptive statistics for the questions

The results revealed a high level of awareness regarding emergency numbers, with 98.25% of correct answers. Similarly, 82.46% demonstrated awareness of cardiac arrest recognition. However, knowledge gaps were identified in areas such as the proper rate of compressions during CPR, where only 35.09% answered correctly. In contrast, a majority (65.79%) displayed knowledge of the recommended compressions-ventilation ratio. Additionally, most respondents (94.74%) correctly knew when to use an AED, while 79.28% identified the contraindications to AED use. In terms of AED-related knowledge, 77.19% knew what an AED is and that they are available in public spaces, but only 1.75% reported personal experience using an AED. Regarding preparedness, 58.77% expressed their readiness to use an AED, while 71.05% affirmed their willingness to perform CPR regardless of their relationship with the victim. A smaller percentage (7.01%) would perform CPR only if they knew the person, and 21.92% indicated a lack of preparedness to perform CPR.

**Fig. 1:**
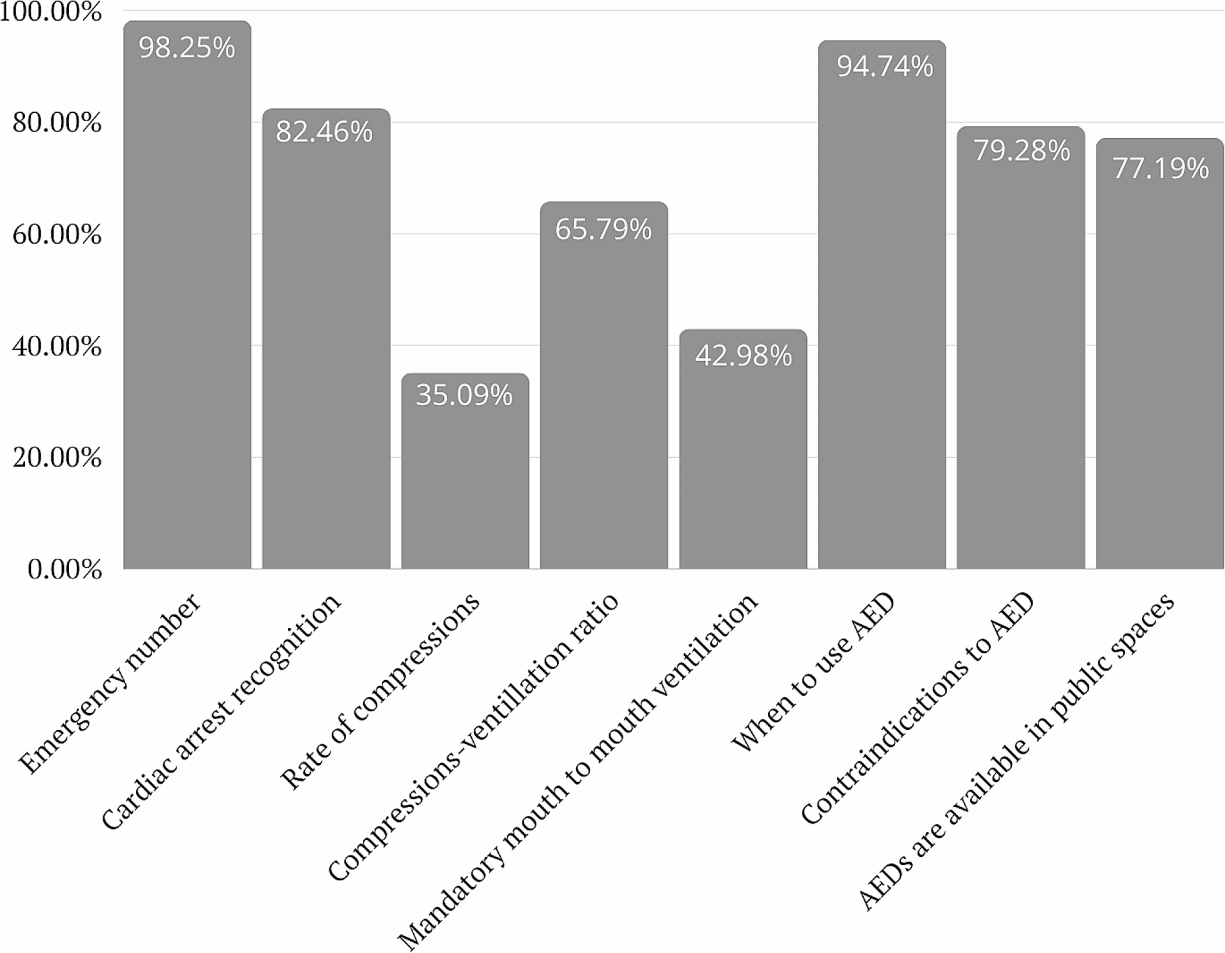
Percentage of correct answers in sections *Basic Life Support Knowledge* and *Automated External Defibrillators* of the survey

**Fig. 2:**
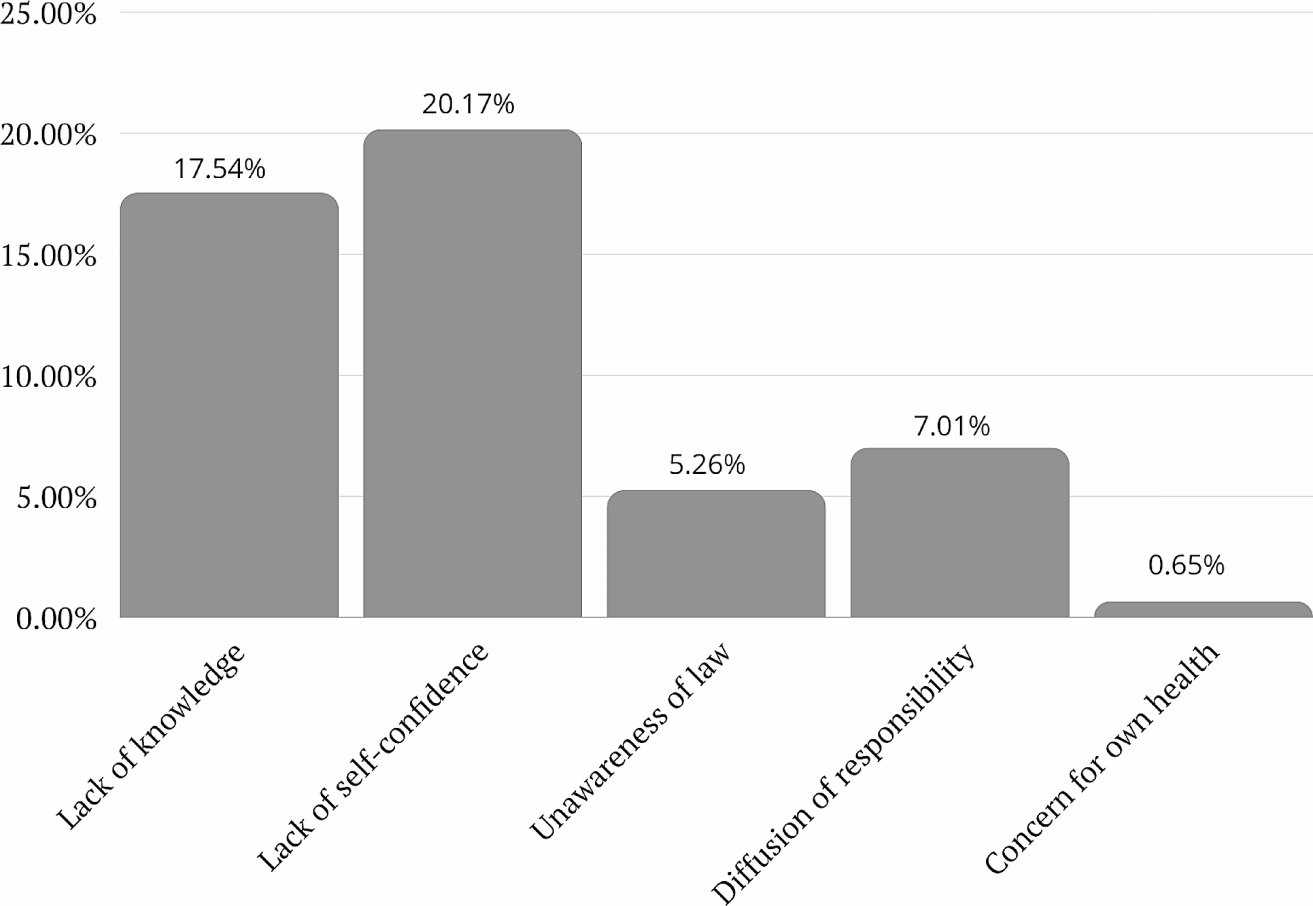
Percentage of answers in section *Personal Attitude* of the survey

A total of 268 participants (58.77%) expressed their preparedness to use an AED. The majority of respondents (324 participants, 71.05%), expressed their readiness to perform CPR. The most common reasons cited were a lack of knowledge (17.54%), lack of self-confidence (20.17%), lack of legal knowledge (5.26%), and diffusion of responsibility (7.01%). Details were presented in Table 3. Figures 1 and 2 represent data from Table 3.

**Table 3:** Detailed distribution of answers to given questions

Problem raised in the question		*n*	%
*Basic CPR knowledge*			
Emergency number		448	98.25
Cardiac arrest recognition		376	82.46
Rate of compressions		160	35.09
Compressions-ventilation ratio		300	65.79
Mandatory mouth to mouth ventilation		196	42.98
*Basic AED knowledge*			
When to use AED		432	94.74
Contraindications to AED		364	79.28
I have used an AED		8	1.75
AEDs are available in public space		352	77.19
*Attitudes towards providing CPR*			
Preparedness to use AED		268	58.77
Preparedness to perform CPR		356	78.06
Reasons for unpreparedness			
	lack of knowledge	80	17.54
	lack of self-confidence	92	20.17
	unawareness of law	24	5.26
	diffusion of responsibility	32	7.01
	concern for own health	3	0.65

#### First aid training

Among the participants, a significant proportion (27.19%, *n* = 124) reported never having received any first aid training. The distribution of participants based on the time since their last training is as follows: 29.82% (*n* = 136) received training more than 10 years ago, 14.91% (*n* = 68) received training 3–5 years ago, 12.28% (*n* = 56) received training 2–3 years ago, 9.65% (*n* = 44) received training 5–10 years ago, and 6.14% (*n* = 28) received training in the current year.

A majority (46.49%, *n* = 212) reported that their first aid training primarily consisted of theory-based approaches, such as presentations and lectures. On the other hand, a smaller proportion (23.68%, *n* = 108) indicated that their training predominantly involved practical components, including hands-on training.

A statistically significant difference was found between first aid training and readiness for performing CPR (*p* = 0.005).

### Knowledge and readiness to perform CPR and use AED

The median number of correct answers in the group of respondents who declared readiness to perform CPR was 5 [4–6]. The median number of correct answers in the group of respondents who did not declare was 4 [3–4]. However, this difference was not statistically significant (*p* = 0.152).

In the group of individuals prepared to use AED, the median of correct answers was 5 [5–6], while in the group of individuals without such a declaration it was 4 [4–5]. This difference was statistically significant (*p* = 0.001).

### Health status and readiness to perform CPR

Individuals who were willing to perform CPR also declared better health (4 [3–4] points in Likert scale) compared to those who were not willing to do CPR (3 [3–4] points in Likert scale). Statistical analysis revealed a significant difference between those groups (*p* = 0.039).

Furthermore, statistically significant differences were found in the willingness to perform CPR among participants suffering from heart failure (*p* = 0.001), anemia (*p* = 0.027), depression (*p* = 0.015), atherosclerosis (*p* = 0.049) and diabetes (*p* = 0.001).

## Discussion

Cardiac arrest is a critical healthcare emergency, with a significant number of cases occurring outside of the hospital setting. Consequently, our societies rely heavily on bystanders to initiate resuscitation before healthcare services arrive [11]. However, considering the rate of bystander CPR ranging from 30 to 40%, it is essential to comprehend the barriers and limitations within society that may hinder the improvement of these numbers [12]. Previous studies have primarily focused on assessing readiness to perform CPR and knowledge regarding CPR and AED use in the general population or among high school/college/university students, with studies focusing on elderly population being a minority [10, 13]. Moreover, it has been observed that increased age is associated with decreased readiness to offer help and a lower frequency of initiating CPR compared to younger bystanders, elderly laypersons were also found to perform worse dispatcher-assissted CPR than younger [5, 10, 14, 15]. These findings, coupled with previously established barriers to successful CPR, such as physical limitations, places the elderly population in an unfavorable position when it comes to performing CPR [8, 16, 17]. To the best of our knowledge, this study represents the first attempt to assess the knowledge and attitude towards performing resuscitation within the elderly population in Poland.

The average lifespan is longer in women in many countries, including Poland, which may account for the predominance of female participants in our study [18]. This gender difference may also play a role in influencing the potential for bystander CPR, as women are reported to feel less comfortable performing CPR, despite not being associated with lower knowledge or readiness levels [10].

The rate of correct answers in both the basic CRP knowledge section and basic AED knowledge section was generally high, ranging from 65.79% for the question about compressions-ventilation ratio to 98.25% for the question about the emergency number. Two exceptions were observed, which included the question about compression rate and the requirement for mouth-to-mouth ventilation. Interestingly, a study conducted in Germany reported similar findings, with compression rate answers performing the least accurately [15]. The poor knowledge regarding ventilation, in our opinion, may have been due to the fact that the study was conducted during the COVID-19 pandemic. At that time, European Resuscitation Council (ERC) published an updated guideline that focused even more attention on safety and emphasized the role of hands-only CPR. These findings indicate the need for targeted educational interventions to enhance knowledge and confidence in performing resuscitation maneuvers. This way, we can empower seniors to take prompt and effective resuscitation efforts, potentially increasing the likelihood of survival during cardiac emergencies.

Since AED use is recommended by numerous authors and studies including ERC Guidelines, the noticeable difference in the prevalence of using AED compared to performing CPR is an important aspect to investigate [19–22]. This disparity is also evident among the elderly population. In line with our findings, Brinkrolf et al. reported that there is less knowledge and lower readiness for AED use comparing to CPR [15].

It is noteworthy that over half of our participants had either received first aid training more than 10 years ago or had never received it at all. In the available literature, 1 month was established an optimal frequency of CPR training resulting in high-quality CPR skills [23]. Moreover, the retention of knowledge is known to be worse in the older population [24]. Therefore, the reported lack of knowledge and self-confidence from our participants is not surprising. Apart from regular training yielding better results, it is crucial to consider the quality of the initial training itself. Older individuals, when properly trained, have demonstrated their ability to perform effective CPR [5]. Miotto et al. found that a group that received only theoretical training performed consistently worse compared to those who received a mixed theoretical-practical training [25]. Furthermore, receiving feedback was identified as an additional important factor that improved trainees’ outcomes [26]. Interestingly, it was found that the method of hands-on training did not significantly affect outcomes, opening up numerous opportunities for future training approaches [27–30]. It’s also of great importance to mention the need for tailoring courses for the specific needs and limitations of the elderly population [31]. Short training sessions adjusted to varied psychophysical abilities are preferable.

As the Polish population is experiencing rapid aging, it becomes crucial to examine how various health conditions, prevalent among the older population, might impact the ability to perform CPR [32]. It is a physically challenging activity that can cause muscular and physiological stress, fatigue, and ultimately, lower back pain [33]. Chronic diseases frequently lead to a decrease in patients’ quality of life and can also diminish their physical activity levels and overall physical capabilities [9]. For instance, patients suffering from heart failure reported feeling not ready to engage in physical activity [34]. It’s important to consider elderly comorbidities as a factor that can influence the willingness and readiness to perform CPR, and this topic should be also targeted during CPR courses [8].

### Limitations

This study has several limitations. We chose UTA because of the good availability of respondents and easy distribution of questionnaires. However, the sample of respondents may not be fully representative of the entire elderly population. Certain groups could be overrepresented or underrepresented, affecting the generalizability of the findings. Furthermore, in questions regarding willingness to perform CPR respondents might provide answers that they perceive as socially acceptable or desirable, rather than their true beliefs or behaviors.

## Conclusions

A wide access to regular CPR training should be ensured for elderly. During training, special attention should be paid to the recognition of cardiac arrest and the details of the quality of the compressions. Participants with cardiovascular disease in particular should be encouraged to perform CPR. Future research should continue to explore and address the barriers to bystander CPR among this population, with the ultimate goal of improving survival rates during OHCA.

### Electronic supplementary material

Below is the link to the electronic supplementary material.

**Supplementary Material 1:** Senior Resuscitation Knowledge and Attitudes Survey

## Data Availability

The datasets used and/or analysed during the current study are available from the corresponding author on reasonable request.

## Abbreviations

UTA: University of the Third Age
CPR: Cardiopulmonary resuscitation
AED: Automated external defibrillator
OHCA: Out-of-hospital cardiac arrests
ERC: European Resuscitation Council
