# Supplementary material for: Knowledge and attitudes towards performing resuscitation among seniors - a population-based study
Source: Arch Public Health. 2024 May 8;82:67. doi: 10.1186/s13690-024-01301-9 (PMC11077712; doi:10.1186/s13690-024-01301-9)
Supplement: Supplementary file 1 — Supplementary Material 1: Senior Resuscitation Knowledge and Attitudes Survey [file 13690_2024_1301_MOESM1_ESM.pdf]

Senior Resuscitation Knowledge and Attitudes Survey

Your participation in this survey is entirely voluntary. If you decide to take part, you may withdraw from the survey at any point. Your responses will be confidential and used solely for research. Your identity will be anonymous in any study-related reports or publications. By participating, you allow data analysis and research use of collected information.

Basic Life Support Knowledge

1. What is the correct emergency telephone number for ambulance service in Poland?

- ○ 911
- ○ 997
- ○ 999
- ○ 998

2. When should chest compressions be performed?

- ○ Unconscious person with normal breathing
- ○ Anyone having a heart attack
- ○ Unconscious person without breathing
- ○ Anyone who has fainted

3. What is the correct rate of chest compressions during resuscitation?

- ○ 50-70 compressions per minute
- ○ 100-120 compressions per minute
- ○ 70-100 compressions per minute
- ○ 150-200 compressions per minute

4. What is the correct ratio of chest compressions to rescue breaths during resuscitation?

- ○ 30 compressions : 2 breaths
- ○ 30 compressions : 5 breaths
- ○ 15 compressions : 2 breaths
- ○ 5 compressions : 1 breath

5. Are rescue breaths necessary during resuscitation?

- ○ Yes
- ○ No

Automated External Defibrillators

6. Have you used an Automated External Defibrillator (AED)?

- ○ Yes
- ○ No

7. When should an Automated External Defibrillator be used?

- ○ Yes
- ○ No

8. When should chest compressions be performed?

- ○ Sudden cardiac arrest
- ○ Person with cardiac pacemaker
- ○ Heart attack
- ○ Conscious person after fainting

9. When should an Automated External Defibrillator NOT be used?

- ○ Victim in a water-filled bathtub
- ○ Heart transplant recipient
- ○ Person with cardiac pacemaker implanted
- ○ Person with no history of heart disease

10. Are you willing to use an Automated External Defibrillator if necessary?

- ○ Yes
- ○ No

Personal Attitude

11. Would you perform cardiopulmonary resuscitation if needed?

- ○ Yes
- ○ No

12. If you answered *"No"* to questions 10 or 11, please indicate the reason(s) (multi-answer question)**:**

- ☐ Lack of knowledge
- ☐ Lack of confidence/fear of judgment
- ☐ Age-related limitations
- ☐ Fear of legal liability for incorrect care
- ☐ Concerns about personal health

Epidemiological Information

13. Rate your health condition from 1 (very poor) to 5 (very good):

○ 1 ○ 2 ○ 3 ○ 4 ○ 5

14. Do you have any of the following conditions? (multi-answer question)**:**

- ☐ Rheumatoid arthritis
- ☐ Osteoporosis
- ☐ Vertigo
- ☐ Anemia
- ☐ Depression
- ☐ Arteriosclerosis
- ☐ Urinary incontinence
- ☐ Diabetes mellitus
- ☐ Heart failure

15. Your age: ________

16. Your sex:

- ○ Female
- ○ Male

17. Your education level:

- ○ Tertiary
- ○ Primary
- ○ Secondary
- ○ Basic vocational

18. When did you last complete a first aid training course?

- ○ Never
- ○ 3-5 years ago
- ○ This year
- ○ 5-10 years ago
- ○ 2-3 years ago
- ○ Over 10 years ago

19. What was the form of the last first aid training you completed? (Skip if never trained):

- ○ Mostly theoretical (lectures and demonstrations)
- ○ Mostly practical (hands-on training)

20. Your residence area:

- ○ Countryside
- ○ City 100,001-500,000 residents
- ○ City <20,000 residents
- ○ City >500,000 residents
- ○ City 20,001-100,000 residents

21. Marital status:

- ○ Divorced
- ○ Widowed
- ○ Married
- ○ Single

Thank you for taking the time to complete the survey.
